# Supplementary material for: Genome-Wide Transcriptional Responses of Marine Nematode Litoditis marina to Hyposaline and Hypersaline Stresses
Source: Front Physiol. 2021 May 4;12:672099. doi: 10.3389/fphys.2021.672099 (PMC8129518; doi:10.3389/fphys.2021.672099)
Supplement: Supplementary file 5 [file Data_Sheet_1.docx]

Supplementary Material

# Supplementary Figures and Tables

## Supplementary Figures


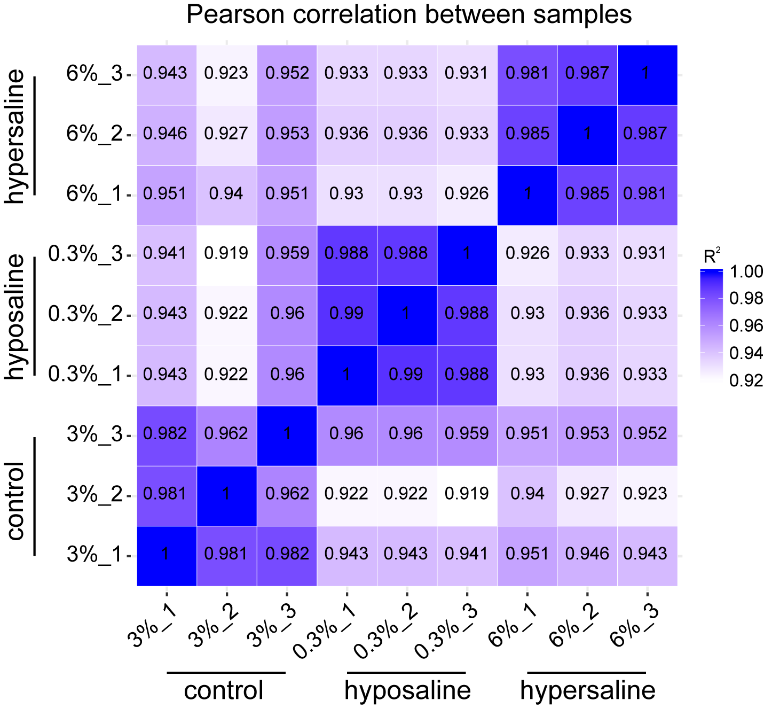


**Supplementary Figure 1**. Heatmap of correlation analysis between samples. The Pearson correlation coefficients (R) of totally nine samples were calculated based on the FPKM value. The scale bar shows the value of R^2^. The high correlation coefficient (> 0.92) within three replicates for each group, indicating reliable sample preparation.


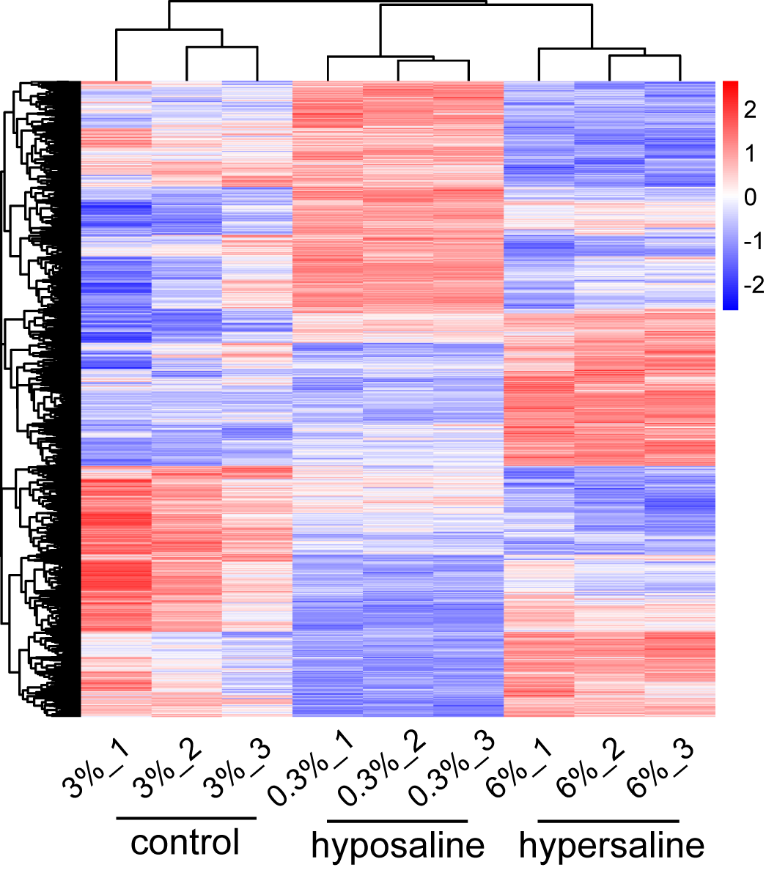


**Supplementary Figure 2**. Heatmap of expression levels for all DEGs among nine samples. The transition from blue to red strips represents an increase in gene expression levels.


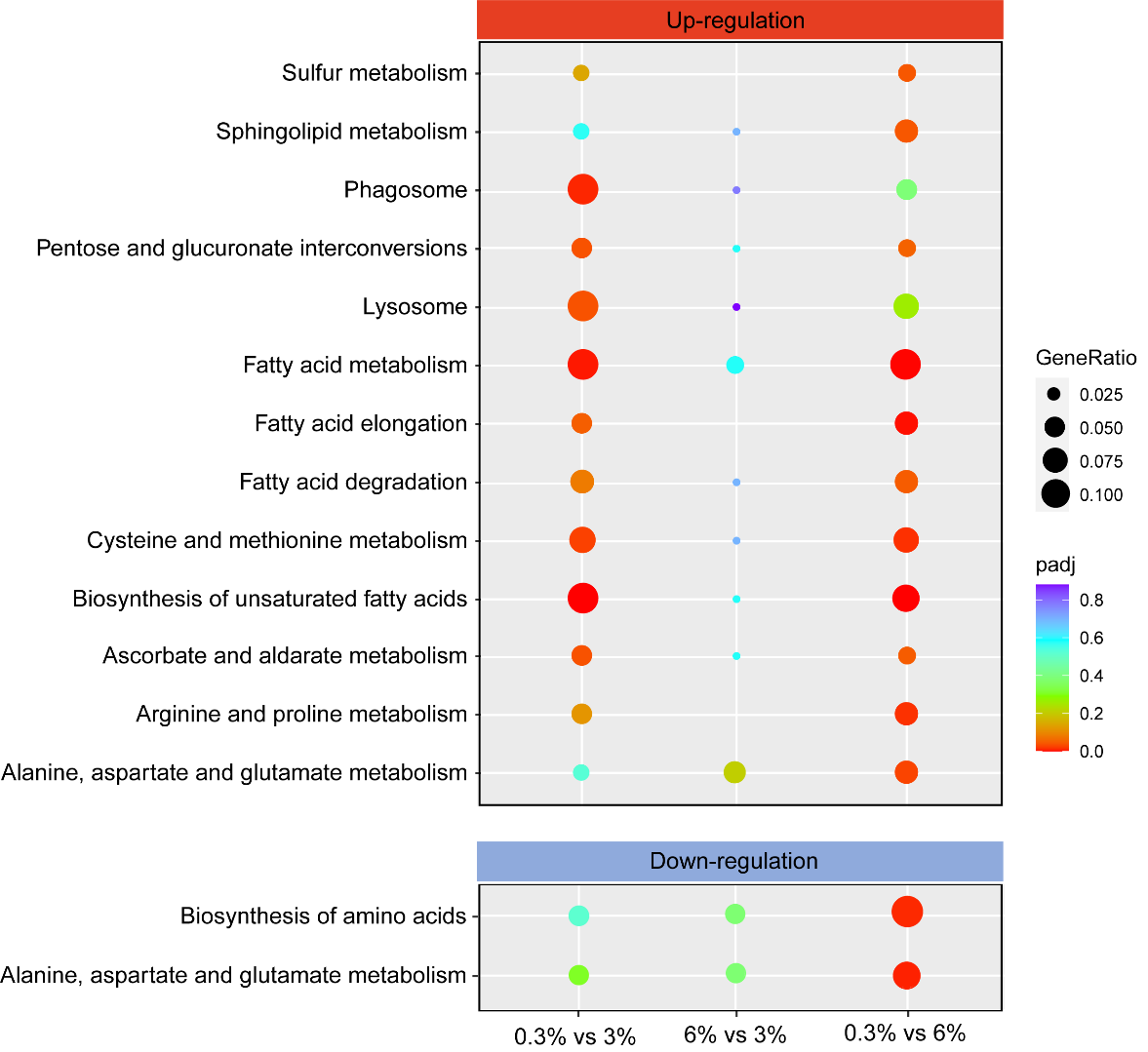


**Supplementary Figure 3.** KEGG pathway enrichment analysis for DEGs. |log2foldchange|>1; DESeq2 padj<0.05 was set as the differential gene screening threshold. KEGG pathway enrichment analysis of DEGs were achieved by clusterProfiler R package (v3.4.4), an adjusted *P*-value (padj) < 0.05 was considered significantly enriched. The color from red to purple represents the significance of the enrichment. GeneRatio was defined as the ratio of the number of differential genes annotated on the KEGG pathway to the total number of differential genes.

-
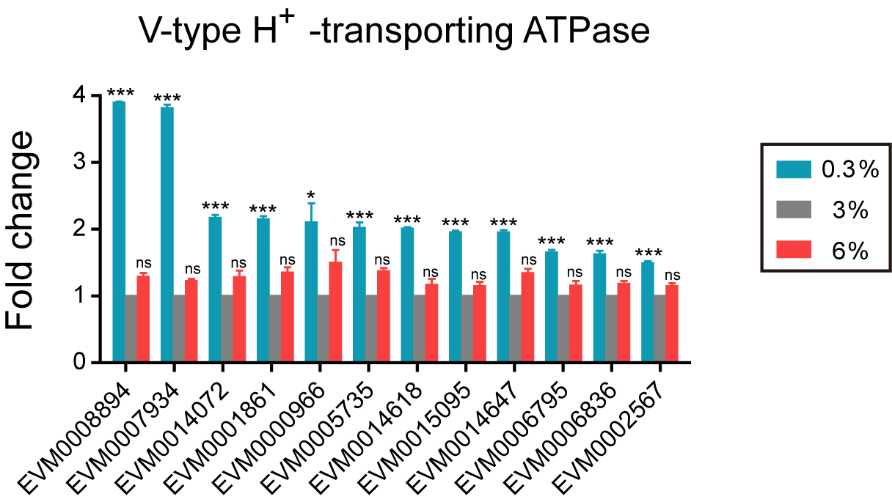


**Supplementary Figure 4.** Expression level of V-type H+-transporting ATPase genes upon salinity stresses in *L. marina*. Fold change indicates the ratio of FPKM value of the treatment group (0.3%, 6%, as indicated) to that of the control group (3%). The error bars represent standard error of the mean of three biological replicates per condition. The comparisons between the treatment and control groups were analyzed statistically using the Benjamini and Hochberg’s methods calculated by DESeq2. The value of padj < 0.05 was considered statistically significant. * padj < 0.05, *** padj < 0.001, ns - not significant.


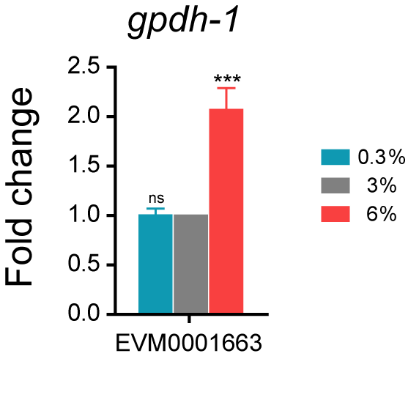


**Supplementary Figure 5.** Expression level of *L. marina* glycerol-3-phosphate dehydrogenase gene, *gpdh-1*, upon salinity stresses. Fold change indicates the ratio of FPKM value of the treatment group (0.3%, 6%, as indicated) to that of the control group (3%). The error bars represent standard error of the mean of three biological replicates per condition. The comparisons between the treatment and control groups were analyzed statistically using the Benjamini and Hochberg’s methods calculated by DESeq2. The value of padj < 0.05 was considered statistically significant. *** padj < 0.001, ns - not significant.


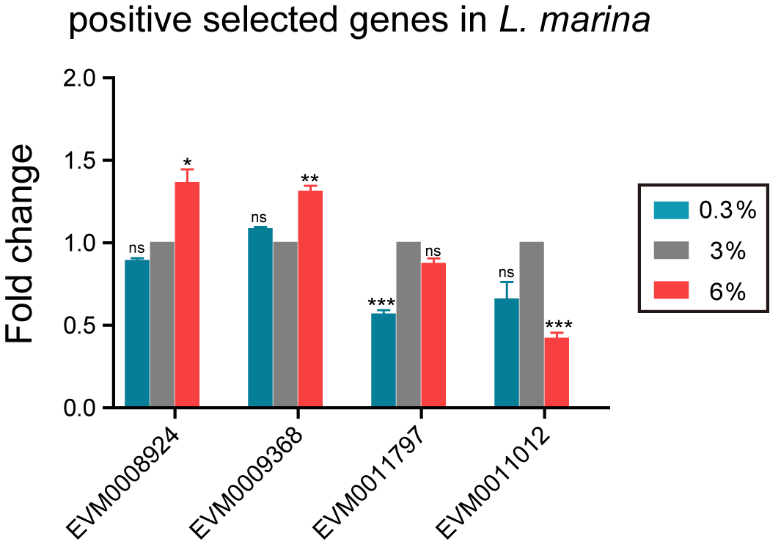


**Supplementary Figure 6.** Expression level of four positive selected genes in *L. marina*, EVM0008924/*imp-2*, EVM0009368/*lec-1*, EVM0011797/*gem-1* and EVM0011012/*T13H5.6*, upon salinity stresses. Fold change indicates the ratio of FPKM value of the treatment group (0.3%, 6%, as indicated) to that of the control group (3%). The error bars represent standard error of the mean of three biological replicates per condition. The comparisons between the treatment and control groups were analyzed statistically using the Benjamini and Hochberg’s methods calculated by DESeq2. The value of padj < 0.05 was considered statistically significant. * padj < 0.05, ** padj < 0.01, *** padj < 0.001, ns - not significant.

## Supplementary Table

**Supplementary Table 1. Statistics of mapping ratio.**


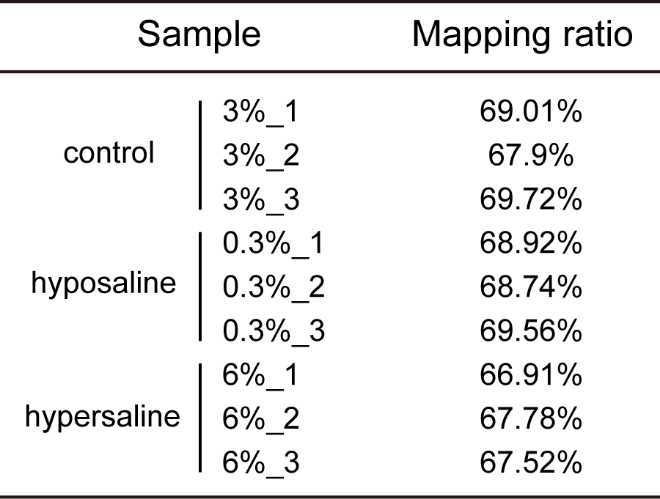


Clean data were aligned to the *L. marina* reference genome by Hisat2 (v2.0.5, with the default parameters). Mapping ratio for each sample were summarized.

# Supplementary files

## Supplementary file 1. Primers information used in this paper.

## Supplementary file 2. Details of significantly up-regulated and down-regulated DEGs identified in salinity stressed worms. |log2foldchange|>1; DESeq2 padj<0.05 was set as the differential gene screening threshold.

## Supplementary file 3. Detailed information for correlation analysis of the results of RNA-seq and qPCR for interest genes.

## Supplementary file 4. A series of heat-shock genes and dozens of proteasome related genes were significantly induced by both low and high salinity stresses.
